# The Role of Neuropeptide B and Its Receptors in Controlling Appetite, Metabolism, and Energy Homeostasis

**DOI:** 10.3390/ijms22126632

**Published:** 2021-06-21

**Authors:** Tatiana Wojciechowicz, Maria Billert, Mariami Jasaszwili, Mathias Z. Strowski, Krzysztof W. Nowak, Marek Skrzypski

**Affiliations:** 1 Department of Animal Physiology, Biochemistry and Biostructure, Poznań University of Life Sciences, 35 Wołyńska St, 60-637 Poznań, Poland; tatiana.wojciechowicz@up.poznan.pl (T.W.); maria.billert@up.poznan.pl (M.B.); mariami.jasaszwili@up.poznan.pl (M.J.); kwnowak@up.poznan.pl (K.W.N.); 2 Department of Hepatology and Gastroenterology, Charité-University Medicine Berlin, D-13353 Berlin, Germany; mathias.strowski@charite.de; 3 Department of Internal Medicine-Gastroenterology, Park-Klinik Weissensee, D-13086 Berlin, Germany

**Keywords:** appetite, neuropeptide B, NPBWR1, NPRBWR2, metabolism, energy homeostasis

## Abstract

Neuropeptide B (NPB) is a peptide hormone that was initially described in 2002. In humans, the biological effects of NPB depend on the activation of two G protein-coupled receptors, NPBWR1 (GPR7) and NPBWR2 (GPR8), and, in rodents, NPBWR1. NPB and its receptors are expressed in the central nervous system (CNS) and in peripheral tissues. NPB is also present in the circulation. In the CNS, NPB modulates appetite, reproduction, pain, anxiety, and emotions. In the peripheral tissues, NPB controls secretion of adrenal hormones, pancreatic beta cells, and various functions of adipose tissue. Experimental downregulation of either NPB or NPBWR1 leads to adiposity. Here, we review the literature with regard to NPB-dependent control of metabolism and energy homeostasis.

## 1. Introduction

Peptides that regulate appetite play a prominent role in controlling energy homeostasis and whole-body metabolism. Such peptides are found in brain regions that are involved in the modulation of appetite. In addition, such peptides are present in the circulation and in numerous peripheral tissues. There is growing evidence indicating that peptides that control appetite (e.g., kisspeptin, orexins, spexin, adropin, apelin, phoenixin, ghrelin, amylin, and pancreatic peptides) also modulate the endocrine activity of endocrine glands as well as lipid and glucose metabolism [1,2,3,4,5,6]. Moreover, some peptides are involved in regulating the endocannabinoid system and, through it, food intake, e.g., hemopressin, a small peptide derived from the α-chain of hemoglobin, reduces appetite through increased levels of endocannabinoids [7,8]. On the other hand, endogenous cannabinoids can also increase the secretion of feeding-regulated hypothalamic neuropeptides [9]. Thus, peptide hormones and their receptors may be of interest in therapy for obesity and obesity-related diseases such as type 2 diabetes [1]. Almost 20 years after the discovery of neuropeptide B (NPB), there is growing evidence that this peptide modulates food intake, body weight, and lipid and glucose metabolism. In our narrative review, we discuss current findings regarding the role of NPB and its receptors in controlling food intake and energy homeostasis.

## 2. Discovery, Structure, and Expression of NPB and Its Receptors

By analyzing human genomic sequences in the Celera database, in 2002, Fuji et al. identified a new neuropeptide composed of 23 or 29 amino acids that was uniquely modified with bromine. This peptide was termed neuropeptide B (NPB) [10]. The same study showed that NPB interacts with NPBWR1 (GPR7) and less potently with NPBWR2 (GPR8) [10]. At the same time, NPB as a ligand of NPBWR1 and NPBWR2 was reported by two independent laboratories [11,12]. Both NPBWR1 and NPBWR2 belong to the G protein-coupled receptor superfamily [13]. It is important to note that humans express both types of receptors, while rodents express only NPBWR1 [14]. It should be pointed out that both types of NPB receptors interact with another ligand, termed neuropeptide W [11,15]. The intracellular signaling of NPBWR1 and NPBWR1 encompasses the modulation of cAMP, calcium, phospholipase C, or MAP kinase signaling [10,11,16,17]. The expression of NPB and its receptors in the CNS and various peripheral tissues was reported (Table 1 and Table 2). 

## 3. The Role of NPB in Appetite Modulation

The initial study showed that NPB mRNA is expressed in brain regions that are crucially relevant in the regulation of food intake. NPB mRNA was reported in the dorsomedial, paraventricular, and arcuate nuclei [11]. In their pioneer work, Tanaka et al. investigated the effects of NPB administration on food intake in mice. Initially, the authors investigated the effects of NPB administration on appetite during the light phase. However, they did not observe any influence of NPB on appetite control in animals. In contrast, i.c.v. administration of NPB during the dark phase led to stimulated food intake during the first 2 h [11]. In contrast, after 2 more hours, NPB caused appetite suppression. The same study evaluated the effects on appetite of co-administration of NPB and corticotropin-releasing factor (CRF), a well-known suppressor of food intake [29]. Tanaka et al. reported that CRF significantly enhanced the suppression of appetite induced by NPB, suggesting an interaction between CRF and urocortin systems [11]. In summary, this study showed, for the first time, that the effects of NPB on food intake are biphasic. 

The anorexigenic activity of the NPB/NPBW1 system was additionally confirmed by Ishii et al., who found that GPR7−/− male mice ate more food than wild-type GPR7 mice [30]. It is worth noting that GPR7−/− mice had reduced NPY mRNA and increased POMC mRNA expression in the hypothalamus. Of note, NPY promotes food intake, while POMC has the opposite effect [31]. Another animal study showed that i.c.v. administration of NPB (during the light phase) in male rats promoted feeding behavior [32]. Stimulation of food intake was detected 30 min after NPB administration and lasted at least 4 h. In contrast, NPB did not affect water intake [32]. It is important to note that, in contrast to NPBW1−/− mice, NPB−/− mice had a normal feeding behavior [33]. Studies addressing the role of NPB in appetite regulation are not restricted to rodents. For instance, it was found that i.p. injection of NPB stimulated mRNA expression of NPY and CCK1 in the hypothalamus of Nile tilapia *Oreochromis niloticus* [34]. Since NPY stimulates food intake and CCK1 suppresses appetite [35], it is difficult to define the role of NPB in controlling feeding behavior in tilapia, and more studies need to be conducted. 

Discussing the contribution of NPB to appetite modulation, it is worth noting that a human study was conducted on circulating NPB in blood in patients with anorexia nervosa (AN). Grzelak et al. reported that patients who suffer from AN are characterized by lower levels of NPB in the circulation compared to healthy controls, suggesting the use of NPB in diagnosing AN [36]. Nevertheless, as pointed out in this work, NPB levels were evaluated in only 30 healthy controls and 46 patients with anorexia [36]; therefore, these results should be interpreted cautiously. The downregulation of circulating NPB levels in patients with anorexia was independently confirmed by a study of 30 healthy controls and 30 patients with AN [37]. Importantly, this study additionally showed that increased NPB levels are not affected by body weight normalization after hospitalization [37]. More studies are needed to elucidate the potential role of NPB in the diagnosis of AN.

In summary, animal studies have shown that i.c.v. administration of NPB during the dark phase biphasically modulates food intake. NPB promotes food intake during the first 2 h, followed by appetite suppression. In contrast, rat studies showed that NPB displays orexigenic effects during the light phase. The role of NPB in controlling feeding behavior is complex; therefore, more studies are needed. 

## 4. The Role of NPB in Brain

Beside its role in feeding behavior, in the CNS, NPB modulates locomotion and analgesia [11]. An i.c.v. injection of NPB in rats significantly increased locomotion in an open-field test in both the bright and dark phases. On the other hand, Hirashima et al. demonstrated that i.c.v. injection of NPB in mice reduced locomotor activity during the dark period, but not during the light phase. The activity of mice was measured using an infrared activity monitor [38]. In experiments using Npb−/− mice, no significant differences in activity levels were found compared to littermate controls [33]. 

In the CNS, NPB impacts analgesia. Tanaka et al. reported that i.c.v. injection of NPB in rats reduced licking duration in the formalin test, which indicates an analgesic role of the peptide against chemically induced pain [11]. These effects could be conferred via NPB and NPBWR1, which are found in the periaqueductal gray matter and amygdala, areas that are also known to express opioid receptors [39]. It is worth mentioning that NPBWR1 binds non-selective opioid ligands such as β-endorphin [13]. The analgesic effect of NPB was also observed after intrathecal injection in the formalin test, and mechanical allodynia was inducible by carrageenan injection [40]. However, NPB had no effect on the level of thermal hyperalgesia induced by paw carrageenan injection in rats [40] and NPB−/− mice [33]. 

The pain response is tightly connected to anxiety [41]. The role of NPB in regulating anxiety has been investigated using the cued and contextual fear test and elevated plus maze test. NPBWR1−/− mice had similar behavior in the contextual fear test compared to wild-type mice [42]. However, unlike wild-type mice, NPBWR1−/− mice showed behavioral changes in social interactions [42]. The role of NPB in the context of social behavior was evaluated by Watanabe et al. [43]. They showed that genetic changes in NPWR1 (single-nucleotide polymorphism at nucleotide 404 resulted in an amino acid change, Y135F) modulated emotional responses to facial expression. The 404AT subjects were less submissive to angry faces than 404AA subjects.

There is evidence that NPB is involved in sleep/wakefulness [38]. An i.c.v. injection of NPB in mice during the dark period decreased time in the waking state and increased time in slow-wave sleep, whereas no change in paradoxical sleep time was observed. Moreover, NPBWR1+/− and NPBWR1−/− mice did not present any abnormalities compared with wild-type mice, indicating a modulatory role of NPB and NPBWR1 in the sleep/wakefulness pattern [38]. 

In summary, NPB plays a role in the regulation of locomotion and decreases locomotor activity during the dark phase. Moreover, during the dark period, NPB decreases the waking state time. It also plays an analgesic role in chemically induced pain and decreases social anxiety.

## 5. The Role of NPB in Regulating Glucose and Lipid Metabolism 

### 5.1. In Vivo Lessons from Genetically Engineered Animals

An animal study performed with NPBWR1−/− mice showed that NPB/NPBWR may play a role in controlling energy hemostasis and body weight regulation. It was reported that depletion of NPBWR1 was accompanied by increased body weight in animals fed a standard or high-fat diet [30]. Notably, this effect was observed only in male animals [30]. An analysis of body composition in male NPBWR1−/− mice showed that total body fat mass was two times higher compared to NPBWR1+/+ animals [30]. Furthermore, male NPBWR1-deficient mice had decreased spontaneous locomotor activity, reduced oxygen consumption, and lower energy expenditure. Additionally, it was found that NPBWR1−/− males at 52 weeks of age had increased blood glucose, leptin, and insulin levels. Sex-dependent effects of NPBWR1 depletion on body weight and metabolic parameters are not completely understood. However, it is unlikely that sex hormones have a role in this context. The contribution of the NPB/NPBWR1 system to body weight control was additionally studied by Kelly et al. using NPB-depleted mice. This study showed that male NPB−/− and NPB+/− mice gained more body weight in response to feeding with low-fat chow (6%) compared with wild-type animals [33]. Overall, these in vivo studies show that the downregulation/depletion of NPBWR1 or NPB leads to an increased body weight, accompanied by decreased energy expenditure. 

### 5.2. The Role of NPB in Controlling Members of the Adipoinsular Axis 

The contribution of the NPBWR1/NPB system to energy homeostasis is additionally supported by in vitro studies suggesting that NPB may interact with fat tissue and pancreatic beta cells. There is growing evidence indicating that energy homeostasis and metabolism are modulated by adipose tissue and by pancreatic beta cells, the exclusive source of insulin [44,45]. Therefore, we assessed the effects of NPB on white adipocytes. First, we found that NPB and its receptor (NPBWR1) mRNA are expressed in isolated rat white preadipocytes and mature adipocytes. Moreover, our study demonstrated that by acting on isolated rat white adipocytes, NPB suppresses leptin mRNA expression and leptin secretion [27]. We also found that NPB stimulates lipolysis and resistin secretion [27]. Overall, these results suggest that NPB may protect against body weight gain by promoting lipolysis. Furthermore, keeping in mind that fat tissue is the main source of leptin, it cannot be excluded that the orexigenic effects of NPB reported by others may result from attenuated leptin production and secretion. In this context, it is worth noting that NPBWR1−/− male mice have elevated levels of leptin in the blood [30]. Nevertheless, it was found that NPB administration in rats had no effect on circulating leptin levels [46]. Thus, the ability of NPB to regulate leptin expression and secretion in vivo is unclear and requires further experimentation. 

In addition to the function of modulating white adipocytes, our recent data indicate that NPB may be involved in brown adipogenesis [28]. We demonstrated that NPB promotes proliferation of rat primary preadipocytes. We also found that NPB acting on rat brown preadipocytes stimulates the expression of adipogenic genes such as PRDM16 and UCP1, which indicates that NPB stimulates the differentiation of brown preadipocytes into mature adipocytes. It is worth noting that the stimulation of UCP1 expression is mediated by p38 kinase activation, the main protein kinase involved in brown adipogenesis [47]. Nevertheless, since our experiments were restricted to in vitro settings only, a question is raised about the physiological consequences of these findings. Importantly, the loss of brown adipose tissue is a hallmark of obesity, while the activation of brown fat in obese individuals leads to body weight loss [48,49]. Therefore, it cannot be excluded that the induction of body weight gain reported in NPB- or NPBWR1-deficient animals resulted from impaired brown adipogenesis. However, the role of the NPB/NPBWR1 system in controlling brown fat tissue formation remains unknown. 

Finally, our recent in vitro study suggested that NPB may modulate glucose hemostasis by controlling insulin secretion. Using insulin-producing rat INS-1E cells and isolated rat pancreatic islets, we found that NPB stimulated insulin mRNA expression and secretion [17]. These results suggest that NPB and its receptors may contribute to glucose control through stimulation of insulin neogenesis and exocytosis. However, it should be pointed out that Rucinski et al. reported a lack of effects of NPB administration on circulating insulin levels in rats [46]. Therefore, the effects of NPB on insulin exocytosis in vivo are not yet fully understood. 

In summary, by acting on mature white adipocytes, NPB promotes lipolysis while suppressing leptin expression and secretion. Furthermore, NPB promotes brown adipogenesis and insulin expression, and secretion in vitro. The effects of NPB on insulin expression and secretion in vivo are yet to be discovered. 

### 5.3. Adrenal Glands

The adrenal glands play a pivotal role in the endocrine system. They mainly produce stress hormones and thus are involved in maintaining energy homeostasis and adaption of the organism to environmental changes [50]. Several research groups have investigated the role of the NPB/NPBWR1 system in the adrenal glands. First, it was found that NPB and NPBWR1 mRNA were expressed in freshly dispersed and 4-day cultured rat zona fasciculata/reticularis (ZF/R) cells [26]. The expression of NPB and NPBWR1 mRNA in rat zona glomerulosa (ZG) and ZF/R cells was confirmed by an independent study, which additionally showed that both genes were expressed in the adrenal medulla (AM) [51]. The expression of NPB and NPBWR1 and NPBWR2 mRNA was detected in ZG and ZF/R cells of adrenal cortexes [4]. The same study showed that NPB failed to modulate basal aldosterone secretion from zona granulosa cells [26]. In contrast, NPB potentiated ACTH-stimulated aldosterone secretion. However, NPB was unable to affect cortisol release from ZF/R cells. Furthermore, it was reported that NPB is able to promote ZF/R cell proliferation [26]. The contribution of NPB to the modulation of adrenal gland functions was confirmed by an in vivo study. Samson et al. reported that i.c.v. administration of NPB (1 or 3 nmol) stimulated plasma corticosterone levels in male Sprague Dawley rats. Importantly, NPB-stimulated corticosterone levels were attenuated in rats pretreated with antiserum against corticotropin-releasing factor [32]. In addition, Mogi et al. found that the central administration of NPB increased circulating cortisol concentrations in sheep [52]. 

The effects of NPB on adrenal functions were also studied in human tissues. It was found that, consistent with the results of rat studies, NPB was not able to modulate aldosterone secretion from ZG cells [16]. On the other hand, NPB promoted cortisol release from ZF/R cells. Furthermore, it was shown that stimulation of cortisol secretion by NPB depends on activation of adenylate cyclase/PKA and phospholipase C/PKC signaling [16]. Andreis et al. studied the effects of NPB on the proliferation of human carcinoma-derived NCI-H295 cells (a surrogate model of human adrenocortical cells) [53]. The authors of this study demonstrated that both NPB receptors were expressed at the mRNA level [53]. The same study showed that NPB is able to promote NCI-H295 cell proliferation and suppress apoptosis [53]. The mitogenic action of NPB in NCI-H295 cells was mediated by tyrosine kinase-dependent ERK1/2 activation [53]. Of note, NPB failed to affect the secretion of cortisol or aldosterone from these cells. Hochol et al. studied the effects of NPB on adrenal gland functions using adrenal medulla-containing adrenal quarters [51]. Interestingly, in contrast to previously published data, this study showed that NPB downregulated ACTH-stimulated aldosterone secretion as well as basal and ACTH-stimulated cortisol secretion from adrenal quarters containing cortical and medullary tissues [51]. The same study showed that acute s.c. administration of NPB did not modulate circulating levels of ACTH or aldosterone but increased corticosterone. The role of NPB in controlling the HPA axis was additionally studied in sheep. It was found that i.c.v. injection of NPB (0.5 nmol) increased the cortisol concentration in the blood [52]. In summary, these results collectively show that NPB and its receptors are expressed in the cortex and medulla of adrenal glands. NPB is also implicated in controlling cortisol and corticosterone secretion.

## 6. The Role of NPB and Its Receptors in Reproduction

Few studies have addressed the role of NPB and its receptors in the regulation of reproductive functions. In the rat CNS, the expression of NPB mRNA was detected in the hypothalamus [10,22], which is known as the crucial center of the reproductive hypothalamus–pituitary–gonadal regulatory axis [54]. More detailed analysis showed that, in the hypothalamus, NPB-immunoreactive cells are present in the paraventricular nucleus (PVN), ventromedial hypothalamic nucleus (VMH), dorsomedial hypothalamic nucleus (DMH), and arcuate nucleus (ARC) [55]. Many regions within the hypothalamus also have high levels of NPBWR1 mRNA expression [55]. Of note, VMH activity is involved in sexual behavior, while ARC is importantly involved in GnRH and prolactin release from the anterior pituitary, LH surge, lactation, and growth hormone release [56]. 

Although NPB-expressing neurons were identified in rats [10,22], there is no information regarding sex differences with respect to the occurrence of NPB neurons in other mammals. However, importantly, Ishi et al. demonstrated that NPBWR1-deficient male mice exhibited a sex-specific phenotype of adult-onset obesity [30]. Additional studies showing the importance of NPB in reproduction and the expression of NBP in the CNS of medaka teleost fish (*Oryzias latipes*) were published by Kikuchi et al. [20] and Hiraki-Kajiyma et al. [19]. Both studies confirmed that NPB is preferentially expressed in the female medaka brain, in populations of Vs/Vp and PMm/PMg neurons, whose expression is known to be estrogen-dependent and associated with female sexual receptivity [18]. NPB neurons in PMm/PMg regions are critically dependent on estrogen [20]. Behavioral studies indicated that NPB is a direct mediator of estrogen action in female mating behavior, acting in a female-specific and reversible manner [19]. Moreover, Hiraki-Kajiyma et al., using NPB-deficient medaka, showed that NPB/NPBWR2 signaling is involved in female sexual receptivity. The female-specific neurons located in PMm/PMg neurons are found in the region that is considered homologous to SON/PVN in the mammalian brain. It is possible that the role of NPB in female sexual receptivity may be conserved across vertebrates; however, this hypothesis needs to be investigated. 

Interesting data were obtained in non-mammalian vertebrates, using the chicken as a model, to examine the functionality of the NPB and NPW system and its interaction with the pituitary gland [21]. It was found that NPB mRNA is widely expressed in chicken tissues, including the hypothalamus, while chicken NPB receptor isoforms cNPBWR1 and cNPBWR2 were predominantly expressed in the brain and pituitary. One study confirmed NPB immunoreactivity detected in a population of cells in the rat anterior pituitary [57]. In another study, the mRNA expression of NPB and its two receptors was detected in the rat anterior pituitary gland [24]. Intermediate levels of NPBWR1 and NPBWR2 expression in the rat pituitary gland were also detected [12]. In vivo, i.c.v. injection of NPB into the lateral cerebral ventricle of male rats increased prolactin but decreased GH in the circulation [32]. 

mRNA expression of NPB was detected in peripheral reproductive glands including the ovary, uterus, placenta, testes, and mammary gland in rats [10,12]. Immunohistochemical detection of NPB in reproductive peripheral glands including ovarian thecal cells, granulosa and lutein cells, and oocytes and in Leydig cells of the testes was also reported [24]. For the first time, the functional role of NPB in the regulation of gonadal hormone secretion was analyzed in pigs [23]. Yang et al. confirmed NPB mRNA expression in porcine tissues such as the ovary and the testes, and NPBWR1 and NPBWR2 expression was detected in Leydig cells and ovarian granulosa cells. In vitro experiments showed that NPB promoted testosterone secretion in cultured Leydig cells in a dose-dependent manner. Different doses of NPB also upregulated progesterone secretion in primary cultures of ovarian pig granulosa cells. Low concentrations of NPB (10^−8^ and 10^−10^ M) increased estradiol secretion, but a higher dose (10^−6^ M) inhibited estradiol secretion, in granulosa cell cultures. The identification of the direct effects of NPB on steroid hormone secretion in pig ovarian and testicular cells suggests that the NPB/NPBWR1 or NPBWR2 system plays a role in modulating reproductive functions. NPB, similar to NPW, may play a role in regulating the rat endocrine system via ACTH/steroids. Hochol et al. showed that i.p. bolus injection of NPB or NPW increased plasma levels of parathyroid hormone, corticosterone, and testosterone. NPB also increased the blood concentration of thyroxine [24].

In summary, there are data suggesting that NPB and its receptors are involved in reproductive and sexual behavior functions. Nowadays, data collected from different and distant species of vertebrates indicate that neuropeptide B is an important regulator of the hypothalamic–pituitary–gonadal axis.

## 7. Other Effects of NPB

**Figure 1:**
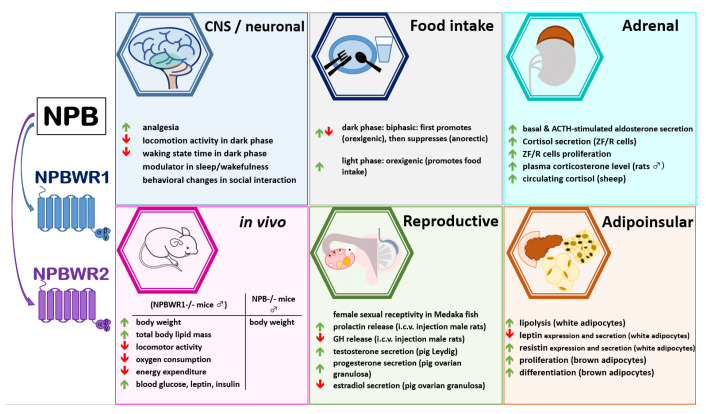
Biological effects of neuropeptide B.

NPB may contribute to the physiology of bones. Ziolkowska et al. reported that NPB and NPBWR1 were expressed in cultured rat calvarial osteoblast-like (ROB) cells [58]. The same study showed that NPB suppressed the proliferation of these cells while it had no effect on osteocalcin secretion [58]. These results indicate that NPB may contribute to the modulation of bone cell activity. In addition, discussing the role of NPB in peripheral tissues, it needs to be pointed out that a recent rat study demonstrated that NPB and its receptors are present in the heart [25]. NPB mRNA expression was detected in both atria and, at lower levels, both ventricles. Importantly, NPB was detected in the bodies of intracardiac ganglion neurons, suggesting that it may be considered as a neurotransmitter. The presence of NPB was also detected in nerve fibers, nerve cell bodies, and smooth muscle in the heart and ganglia. However, the authors of this study failed to detect NPB in cardiomyocyte cells. Due to limited data regarding the role of NPB in the cardiovascular system, it is difficult to speculate about its role in the heart. Nevertheless, based on the presence of NPB in smooth muscle cells of the coronary circulation, it was postulated that it may be involved in modulating the regulation of the coronary circulation [25]. Moreover, NPB expression was also detected in the intestine [10,18,21,23], suggesting its potential role in controlling the gut–brain axis. Nevertheless, more experiments are needed to answer this point. In summary, NPB and its receptors may be involved in controlling bone cell and cardiovascular system functions. As shown in Table 1, NPB is widely expressed in numerous peripheral tissues. The biological effects of neuropeptide B are shown in Figure 1.

## 8. Concluding Remarks

Almost two decades after the discovery of NPB, there is growing evidence indicating its impact on appetite, reproduction, and a variety of endocrine activities in peripheral endocrine glands. Furthermore, studies of genetically engineered animals have shown us that NPB or NPBWR1 depletion is accompanied by increased body weight. In addition, recent data show that NPB may be positively involved in controlling insulin synthesis and secretion as well as promoting brown adipogenesis, which are important targets for therapy in diabetes and obesity. There is also evidence that NPB may be involved in controlling cardiovascular system and adrenal gland functions. These results suggest that NPB and its receptors should be considered in the development of therapy for human diseases such as obesity and type 2 diabetes. 

## Figures and Tables

**Table 1:** Localization of NPB in central nervous system and peripheral tissues.

	RT-PCR	ISH	IHC ICC IF	WB	NB	References
Whole brain	F	-	-	F	-	[18,19]
Telencephalic area (Vs/Vp)	-	F	F	-	F	[18,19]
Magnocellular/gigantocellular portions of magnocellular preoptic nucleus (PMm/PMg)	-	F	F	-	F	[18,19,20]
Telencephalon	Ch	-	-	-	-	[21]
Cerebral cortex	Rt	-	-	-	-	[10]
Striatum	Rt	-	-	-	-	[10]
Hippocampus	Rt, P	M, Rt	-	-	-	[10,11,22,23]
Thalamus	Rt	-	-	-	-	[10]
Hypothalamus	Rt, Ch	-	Rt	-	-	[10,21,24]
Midbrain	Rt, Ch	-	-	-	-	[10,21]
Cerebellum	Rt, P, Ch	-	Rt	-	-	[10,21,23,24]
Medulla oblongata	Rt	-	-	-	-	[10]
Spinal cord	Rt, P, Ch	-	F	-	-	[10,19,21,23]
Lateral habenular nucleus (LHb)	-	M	-	-	-	[11]
Paraventricular hypothalamic nucleus (PVN)	-	M	Rt	-	-	[11,24]
Edinger–Westphal nucleus	-	M, Rt	-	-	-	[22,24]
Motor root of trigeminal nerve (m5)	-	M	-	-	-	[11]
Sensory root of trigeminal nerve (s5)	-	M	-	-	-	[11]
Lateral parabrachinal nucleus internal part (LPBI)	-	M	-	-	-	[11]
Mesencephalic trigeminal nucleus (Me5)	-	M	-	-	-	[11]
Subcoeruleus nucleus alpha part (Sub CA)	-	M	-	-	-	[11]
Locus coeruleus (LC)	-	M, Rt	-	-	-	[11,22]
Noradrenergic cell group A5	-	M	-	-	-	[11]
Interior olive subnucleus B (OIB)	-	M	-	-	-	[11]
Anterior olfactory nucleus	-	Rt	-	-	-	[22]
Piriform cortex	-	Rt		-	-	[22]
Supraoptic nucleus (SON)	-	-	Rt	-	-	[24]
Median preoptic nucleus	-	Rt	-	-	-	[22]
Basolateral amygdala	-	Rt	-	-	-	[22]
Medial tuberal nucleus	-	Rt	-	-	-	[22]
Substantia nigra	-	Rt	-	-	-	[22]
Dorsal raphne nucleus	-	Rt	-	-	-	[22]
Pituitary gland	Rt, Ch	-	Rt	-	-	[10,21,24]
Eyeball and optic nerve	Rt, F (eye)	-	-	-	-	[10,18]
Gill	F	-	-	-	-	[18]
Thyroid gland	Rt	-	Rt	-	-	[10,24]
Trachea	Rt	-	-	-	-	[10]
Thymus	Rt, P	-	-	-	-	[10,23]
Tonsil	P	-	-	-	-	[23]
Heart	Rt. Ch	-	Rt	Rt	-	[10,21,25]
Lung	Rt, Ch	-	-	--	-	[10,21]
Liver	Rt, Ch, F	-	-	-	-	[10,18,21]
Spleen	Rt, Ch	-	-	-	-	[10,21]
Lymph node	Rt	-	-	-	-	[10]
Pancreas	Rt, Ch	-	Rt	-	-	[10,21,24]
Kidney	Rt, Ch	-	-	-	-	[10,21]
Adrenal gland (adrenal medulla, adrenal cortex: zonae glomerulosa and fasciculata/reticularis)	Rt	-	Rt	-	-	[10,24,26]
Urinary bladder	Rt	-	-	-	-	[10]
Peritoneum	Rt	-	-	-	-	[10]
Stomach	-	-	-	-	-	[10]
Duodenum, jejunum, ileum, cecum, colon, rectum	Rt, P, Ch	-	-	-	-	[10,21,23]
Intestine	F	-	-	-	-	[18]
Skeletal muscle	Rt, Ch	-	-	-	-	[10,21]
Prostate	Rt	-	-	-	-	[10]
Seminal vesicle	Rt	-	-	-	-	[10]
Testes	Rt, P, Ch, F	-	Rt	-	-	[10,18,21,23,24]
Ovary	Rt, P, Ch, F	-	Rt	-	-	[10,18,21,23,24]
Uterus	Rt	-	-	-	-	[10]
Placenta	Rt	-	-	-	-	[10]
Mammary gland	Rt	-	-	-	-	[10]
Skin	Rt, Ch	-	-	-	-	[10,21]
Femur	Rt	-	-	-	-	[10]
Bone marrow	Rt	-	-	-	-	[10]
Costal cartilage	Rt	-	-	-	-	[10]
White adipose tissue	Rt, Ch	-	-	-	-	[10,21,27]
Brown adipose tissue	Rt	-	-	-	-	[10,28]
Fetus	Rt	-	-	-	-	[10]
Embryo	F	F	-	-	-	[18]

RT-PCR, real-time PCR; ISH, in situ hybridization; ICH, immunohistochemistry; ICC, immunocytochemistry; IF, immunofluorescence; Rt, rat; M, mouse; H, human; P, pig; Ch, chicken; F, medaka fish.

**Table 2:** Localization of NPBWR1 (R1) and NPBWR2 (R2) in central nervous system and peripheral tissues.

	RT-PCR	ISH	IHC ICC IF	WB	Reference
Telencephalon	Ch (R1, R2)	F (R2)	-	-	[19,21]
Cerebral cortex	Rt (R1)	-	-	-	[10]
Striatum	Rt (R1)	-	-	-	[10]
Hippocampus	Rt (R1)	M	-	-	[10]
Thalamus	Rt (R1)	F (R2)	-	-	[10,19]
Hypothalamus	Rt (R1), Ch (R1, R2)	Rt (R1), F (R2)	-	-	[10,19,21,22,24]
Midbrain	Rt (R1), Ch (R1, R2)	F (R2)	-	-	[10,19,21]
Cerebellum	Rt (R1)	-	-	-	[10]
Medulla oblongata	Rt (R1)	-	-	-	[10]
Amygdala	-	Rt (R1)	-	-	[22]
Suprachiasmatic nucleus	-	Rt (R1)	-	-	[22]
Ventral tuberomammillary nucleus	-	Rt (R1)	-	-	[22]
Dorsal endopiriform	-	Rt (R1)	-	-	[22]
Dorsal tenia tecta	-	Rt (R1)	-	-	[22]
Bed nucleus	-	Rt (R1)	-	-	[22]
Red nucleus	-	Rt (R1)	-	-	[22]
Parastrial nucleus	-	Rt (R1)	-	-	[22]
Laterodorsal tegmentum	-	Rt (R1)	-	-	[22]
Superior colliculus	-	Rt (R1)	-	-	[22]
Locus coeruleus	-	Rt (R1)	-	-	[22]
Nucleus of solitary tract	-	Rt (R1)	-	-	[22]
Spinal cord	Rt (R1), Ch (R1, R2)	F (R2)	-	-	[10,19,21]
Pituitary gland	Rt (R1), Ch (R2)	F (R2)	-	Ch (R1, R2)	[10,19,21,24]
Eyeball and optic nerve	Rt (R1)	-	-	-	[10]
Thyroid gland	Rt (R1)	-	-	-	[10,24]
Trachea	Rt (R1)	-	-	-	[10]
Thymus	Rt (R1)	-	-	-	[10]
Lung	Rt (R1)	-	-	-	[10]
Heart	Rt (R1)	-	Rt (R1)	Rt (R1)	[25]
Adrenal gland (adrenal medulla, adrenal cortex: zonae glomerulosa and fasciculata/reticularis)	Rt (R1)	-	-	-	[10,24,26]
Stomach	Rt (R1)	-	-	-	[10]
Duodenum, jejunum, ileum, cecum, colon, rectum	Rt (R1), Ch (R1)	-	-	-	[10,21]
Testes	Rt (R1), P (R1, R2)	-	-	-	[10,23,24]
Ovary	Rt (R1), P (R1, R2)	-	-	-	[10,23,24]
Uterus	Rt (R1)	-	-	-	[10]
Placenta	Rt (R1)	-	-	-	[10]
Mammary gland	Rt (R1)	-	-	-	[10]
Skin	Rt (R1)	-	-	-	[10]
Fetus	Rt (R1)	-	-	-	[10]
Pancreas	Ch (R2)	-	-	-	[21]
Spleen	Ch (R2)	-	-	-	[21]
Muscle	Ch (R1)	-	-	-	[21]
Brown preadipocytes	Rt (R1)	-	-	-	[28]
White adipocytes	Rt (R1)	-	-	-	[27]

RT-PCR, real-time PCR; ISH, in situ hybridization; ICH, immunohistochemistry; ICC, immunocytochemistry; IF, immunofluorescence; Rt, rat; M, mouse; H, human; P, pig; Ch, chicken; F, medaka fish.

## Abbreviations

ACTH	adrenocorticotropic hormone
AN	anorexia nervosa
ARC	arcuate nucleus
cAMP	adenosine 3′,5′-cyclic monophosphate
CCK1	cholecystokinin A
CNS	central nervous system
CRF	corticotropin-releasing factor
DMH	dorsomedial hypothalamic nucleus
ERK1/2	extracellular signal-regulated kinase 1/2, tyrosine kinase-dependent
GnRH	gonadotropin-releasing hormone
GH	growth hormone
i.c.v.	intracerebroventricular
LC	locus coeruleus
LH	luteotropic hormone
LHb	lateral habenular nucleus
LPBI	lateral parabrachial nucleus internal part
Me5	mesencephalic trigeminal nucleus
MAP kinases	mitogen-activated protein kinases
NPB	neuropeptide B
NPBWR1 (GPR7)	neuropeptide B/W receptor 1
NPBWR2 (GPR8)	neuropeptide B/W receptor 2
NPW	neuropeptide W
NPY	neuropeptide Y
OIB	interior olive subnucleus B
PKA	protein kinase A
PKC	protein kinase C
PMm/PMg	magnocellular/gigantocellular portion of the magnocellular preoptic nucleus
POMC	proopiomelanocortin
Sub CA	subcoeruleus nucleus alpha part
SON	supraoptic nucleus
ZF/R	zona fasciculata/reticularis
ZG	zona glomerulosa
VMH	ventromedial hypothalamic nucleus
Vs/Vp	telencephalic area
